# Supplementary material for: Secreted IgM modulates IL-10 expression in B cells
Source: Nat Commun. 2024 Jan 5;15:324. doi: 10.1038/s41467-023-44382-w (PMC10773282; doi:10.1038/s41467-023-44382-w)
Supplement: Supplementary file 1 — Supplementary Information [file 41467_2023_44382_MOESM1_ESM.pdf]

Secreted IgM modulates IL-10 expression in B cells

Authors: Shannon Eileen McGettigan[1], Lazaro Emilio Aira[1], Gaurav Kumar[2], Romain Ballet[3,4], Eugene C. Butcher[3,4], Nicole Baumgarth[5,6], Gudrun F. Debes[1,7]*

Affiliations:
1. Department of Microbiology and Immunology, Sidney Kimmel Medical College, Thomas Jefferson University, Philadelphia, PA, 19107
2. Department of Cancer Biology, Sidney Kimmel Medical College, Thomas Jefferson University, Philadelphia, PA, 19107
3. Palo Alto Veterans Institute for Research, Veterans Affairs Palo Alto Health Care System, Palo Alto, CA, USA
4. Laboratory of Immunology and Vascular Biology, Department of Pathology, Stanford University School of Medicine, Stanford, CA, USA
5. Center for Immunology and Infectious Diseases, Dept. Pathology, Microbiology & Immunology, University of California Davis, Davis, CA, USA
6. Department of Molecular Microbiology and Immunology, Bloomberg School of Public Health, Johns Hopkins University, Baltimore, MD, USA
7. Sidney Kimmel Cancer Center, Thomas Jefferson University, Philadelphia, PA, 19107

*Corresponding author: Dr. Gudrun F. Debes; Email: Gudrun.debes@jefferson.edu.

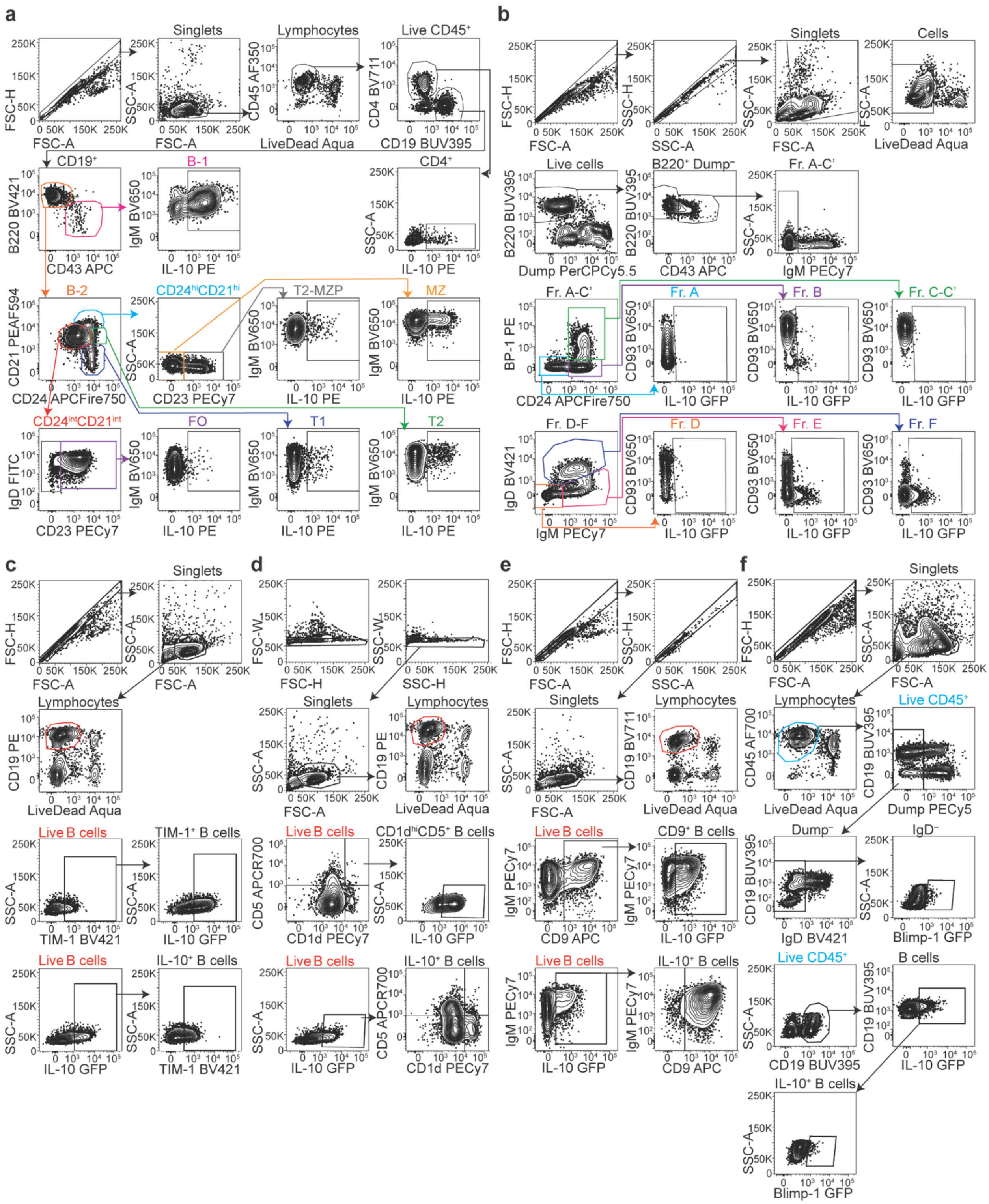
a
Singlets
Lymphocytes
Live CD45+
FSC-H
FSC-A
SSC-A
CD45 AF350
LiveDead Aqua
CD4 BV711
CD19 BUV395
CD19+
B-1
CD4+
B220 BV421
CD43 APC
IgM BV650
IL-10 PE
SSC-A
B-2
CD24hiCD21hi
T2-MZP
MZ
CD21 PEAF594
CD24 APCFire750
CD23 PECy7
CD24intCD21int
FO
T1
T2
IgD FITC
b
Singlets
Cells
FSC-H
SSC-H
SSC-A
FSC-A
LiveDead Aqua
Live cells
B220+ Dump−
Fr. A-C'
B220 BUV395
Dump PerCPCy5.5
CD43 APC
IgM PECy7
Fr. A-C'
Fr. A
Fr. B
Fr. C-C'
BP-1 PE
CD93 BV650
IL-10 GFP
Fr. D-F
Fr. D
Fr. E
Fr. F
IgD BV421
c
Singlets
Lymphocytes
CD19 PE
Live B cells
TIM-1+ B cells
TIM-1 BV421
IL-10 GFP
IL-10+ B cells
d
FSC-W
SSC-W
FSC-H
SSC-H
Singlets
Lymphocytes
Live B cells
CD1dhiCD5+ B cells
CD5 APCR700
CD1d PECy7
IL-10+ B cells
e
Singlets
Lymphocytes
CD19 BV711
Live B cells
CD9+ B cells
IgM PECy7
CD9 APC
IL-10+ B cells
f
Singlets
Lymphocytes
Live CD45+
CD45 AF700
CD19 BUV395
Dump PECy5
Dump−
IgD−
IgD BV421
Blimp-1 GFP
Live CD45+
B cells
IL-10+ B cells

Supplementary Figure 1. Gating strategy for B cell subsets, Bregs, plasma cells, and IL-10 expression.

(a) Flow cytometric gating strategy for B cell subsets and IL-10 expression; related to Figures 1c-f; 2a; 4a-b, e-f, h-I; 5a-b and Supplementary Figures 2c-d; 5b-c. Cells were gated on singlets, lymphocytes, Live/Dead (L/D) stain$^{–}$, CD45^{+}, CD4$^{–}$, CD19^{+}, followed by markers specific to each B cell subset: B-1: B220$^{lo/–}$ CD43^{+}; and B-2: B220^{hi}CD43$^{–}$; B-2 cells further subdivided as transitional-1 (T1): CD24^{hi}CD21$^{lo/–}$; transitional-2 (T2): CD24^{hi}CD21^{int}; transitional-2-marginal zone precursor (T2-MZP): CD24^{hi}CD21^{hi}, CD23^{+}; marginal zone (MZ): CD24^{hi}CD21^{hi} CD23$^{–}$; and follicular (FO): CD24^{int}CD21^{int} CD23^{+}. CD4^{+} T cells were gated on singlets, lymphocytes, L/D stain$^{–}$, CD45^{+}, CD4^{+}, CD19$^{–}$. Markers to delineate subsets were chosen based on those that retained expression equivalently with and without 4-h stimulation with phorbol 12-myristate 13-acetate/ionomycin/lipopolysaccharide (P/I/L). (b) Flow cytometric gating strategy for bone marrow (BM) developing B cells (Figure 2b) assessed after 4-h stimulation with P/I/L for B cell fractions according to the Hardy scheme. Cells were gated on singlets, Live/Dead (L/D) stain$^{–}$ CD3$^{–}$F4/80$^{–}$NK1.1$^{–}$Gr-1$^{–}$B220^{+}. B220^{+} BM cells were further subdivided into Hardy fractions: Fr. A: B220^{+}CD43^{+}IgM$^{–}$BP1$^{–}$CD24$^{–}$; Fr. B: B220^{+}CD43^{+}IgM$^{–}$BP1$^{–}$CD24^{+}; Fr. C-C': B220^{+}CD43^{+}IgM$^{–}$BP1^{+}CD24^{+}; Fr. D: B220^{+}CD43$^{–}$IgM$^{–}$IgD$^{–}$; Fr. E: B220^{+}CD43$^{–}$IgM^{+}IgD$^{–}$; and Fr. F: B220^{+}CD43$^{–}$IgM$^{+/–}$IgD^{+}. (c-e) Flow cytometric gating strategy for splenic Breg markers Figures 2c-e. Breg phenotype identified by expression of TIM-1^{+} (c), CD1d^{hi}CD5^{+} (d), or CD9^{+} (e) among total splenic B cells without stimulation in sIgM$^{–/–}$IL10^{GFP} mice. (f) Flow cytometric gating strategy for Plasma cells (PC), Figure 2f, directly assessed *ex vivo* by L/D stain$^{–}$ CD45^{+}CD3$^{–}$F4/80$^{–}$IgD$^{–}$Blimp-1^{+} lymphocytes using sIgM$^{–/–}$ mice crossed with Blimp1^{GFP} reporter mice (left) and among IL-10^{+} B cells after 4-h stimulation with P/I/L (right).

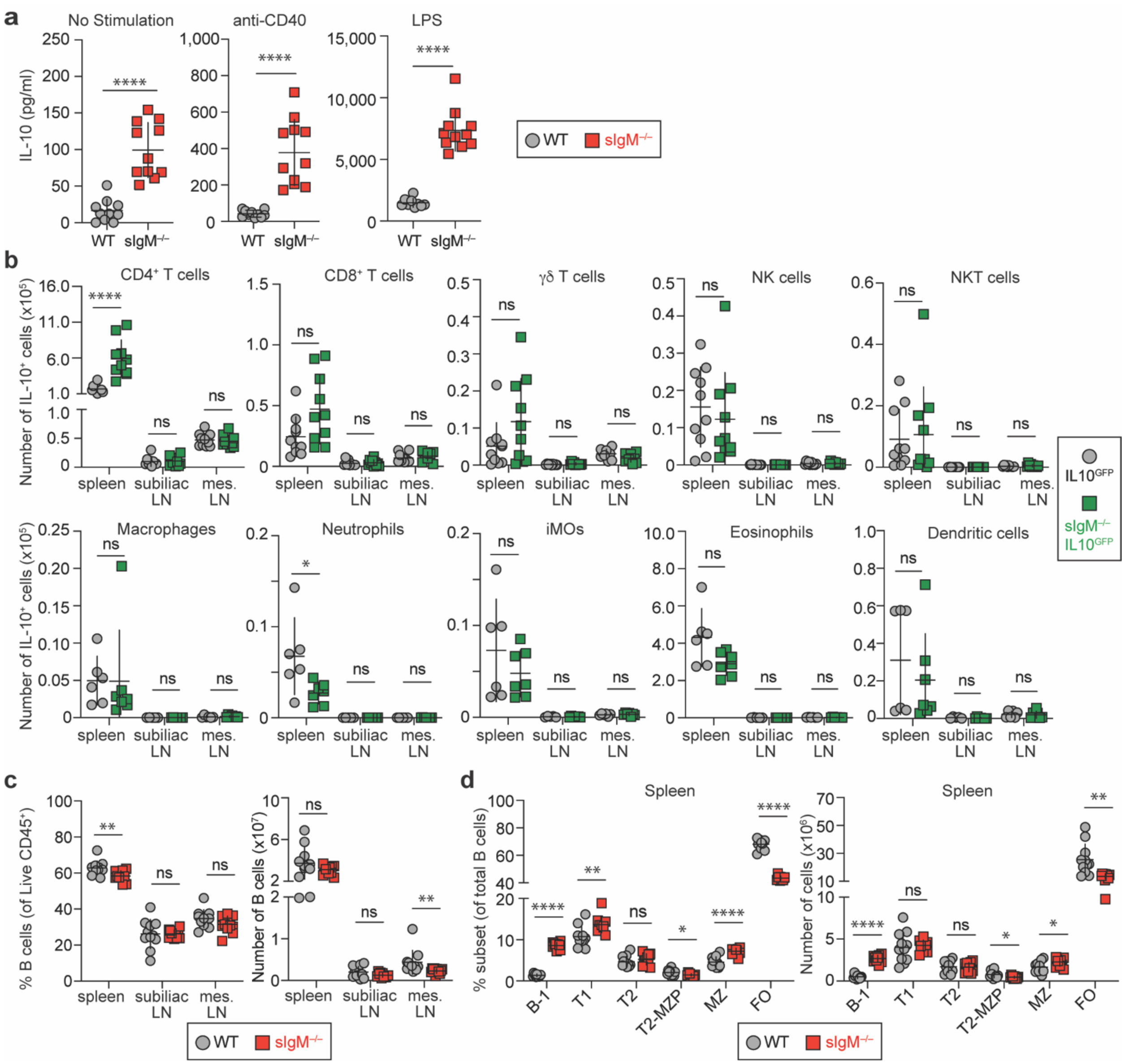

Supplementary Figure 2. Leukocyte IL-10 expression and B cell subsets in sIgM$^{-/-}$ and WT mice.

(a) IL-10 concentration by ELISA in B-cell culture supernatants after 3-day stimulation with 5 μg/ml anti-CD40, 1 μg/ml lipopolysaccharide (LPS), or without stimulation (Nil, WT n=10, sIgM$^{-/-}$ n=11, ****p<0.0001; anti-CD40, WT n=9, sIgM$^{-/-}$ n=11, ****p<0.0001). (b-d) Flow cytometric analysis of leukocyte subsets in spleen, subiliac lymph nodes, and mesenteric (mes.) lymph nodes (LN) of sIgM$^{-/-}$ and WT mice. (b) Frequency of IL-10^{+} cells based on GFP

expression in non-stimulated samples among lymphocyte subsets: pregated on singlets, lymphocyte scatter, LiveDead^{-} CD45^{+} CD19^{-} then subgated for CD4^{+} T cells (CD3^{+}NK1.1^{-}CD4^{+}CD8^{-}), CD8^{+} T cells (CD3^{+}NK1.1^{-}CD4^{-}CD8^{+}), γδ T cells (CD3^{+}NK1.1^{-}γδ TCR^{+}), NK cells (CD3^{-}NK1.1^{+}), NKT cells (CD3^{+}NK1.1^{+}), and myeloid/granulocyte subsets: pregated on singlets, cell scatter, LiveDead^{-} CD45^{+}CD19^{-} then subgated on macrophages (F4/80^{+}), neutrophils (F4/80^{-}CD11b^{+}Ly6G^{+}Ly6C^{+}), inflammatory monocytes (iMOs; F4/80^{-}CD11b^{+}Ly6G^{-}Ly6C^{hi}), eosinophils (F4/80^{-}CD11b^{+}Ly6G^{-}Ly6C^{lo}), and dendritic cells (F4/80^{-}CD11c^{+}). Lymphocyte subsets: Spleen WT n=10, sIgM$^{-/-}$ n=10, subiliac LN WT n=10, sIgM$^{-/-}$ n=10, mes. LN WT n=10, sIgM$^{-/-}$ n=10; CD4^{+} T cells: Spleen ****p<0.0001, subiliac LN p=0.7197, mes. LN p=0.6992; CD8^{+} T cells: Spleen p=0.0630, subiliac LN p=0.8421, mes. LN p=0.4813; γδ T cells: Spleen p=0.2475, subiliac LN p=0.4339, mes. LN p=0.0753; NK cells: Spleen p=0.4359, subiliac LN p=0.3967, mes. LN p=0.5919; NKT cells: Spleen p=0.6842, subiliac LN p=0.6490, mes. LN p=0.6842. Myeloid/granulocyte subsets: Spleen WT n=6, sIgM$^{-/-}$ n=7, subiliac LN WT n=5, sIgM$^{-/-}$ n=7, mes. LN WT n=6, sIgM$^{-/-}$ n=7; Macrophages: Spleen p=0.3660, subiliac LN p=0.5227, mes. LN p=0.5338; Neutrophils: Spleen *p=0.0221, subiliac LN p=0.4167, mes. LN p>0.9999; Inflammatory Monocytes: Spleen p=0.5338, subiliac LN p=0.8674, mes. LN p=0.6282; Eosinophils: Spleen p=0.0734, subiliac LN p=0.2020, mes. LN p=0.3456; Dendritic cells: Spleen p=0.8357, subiliac LN p=0.8763, mes. LN p=0.5338. (c) Frequency (left, WT n=11, sIgM$^{-/-}$ n=11; Spleen **p=0.0032, subiliac LN p=0.9487, mes. LN p=0.2169) and total number (right, WT n=11, sIgM$^{-/-}$ n=11; Spleen p=0.3653, subiliac LN p=0.3080, mes. LN **p=0.0014) of B cells. (d) Frequency and total number of B-1, transitional-1 (T1), transitional-2 (T2), transitional-2 marginal zone precursor (T2-MZP), marginal zone (MZ), and follicular (FO) B cell subsets in the spleen after 4-h stimulation with P/I/L. Frequency,

left, WT n=11, $sIgM^{-/-}$ n=11, B-1 ***p=0.0002, T1 ****p<0.0001, T2 ****p<0.0001, T2-MZP ****p<0.0001, MZ ****p<0.0001, FO ****p<0.0001 and Total Number, right, WT n=11, $sIgM^{-/-}$ n=11, B-1 ****p<0.0001, T1 ****p<0.0001, T2 ****p<0.0001, T2-MZP ****p<0.0001, MZ ****p<0.0001, FO ****p<0.0001. Negligible differences in lymphocyte subset frequencies were noted after 4-h stimulation. Data points indicate mean ± SD and each symbol represents one mouse. P values were calculated using two-tailed Mann-Whitney U test. Data are pooled from 2 (b: myeloid/granulocyte subsets), 3 (b: lymphoid subsets; c; d) or 5(a) independent experiments. Not significant (ns). Source data are provided with this paper.

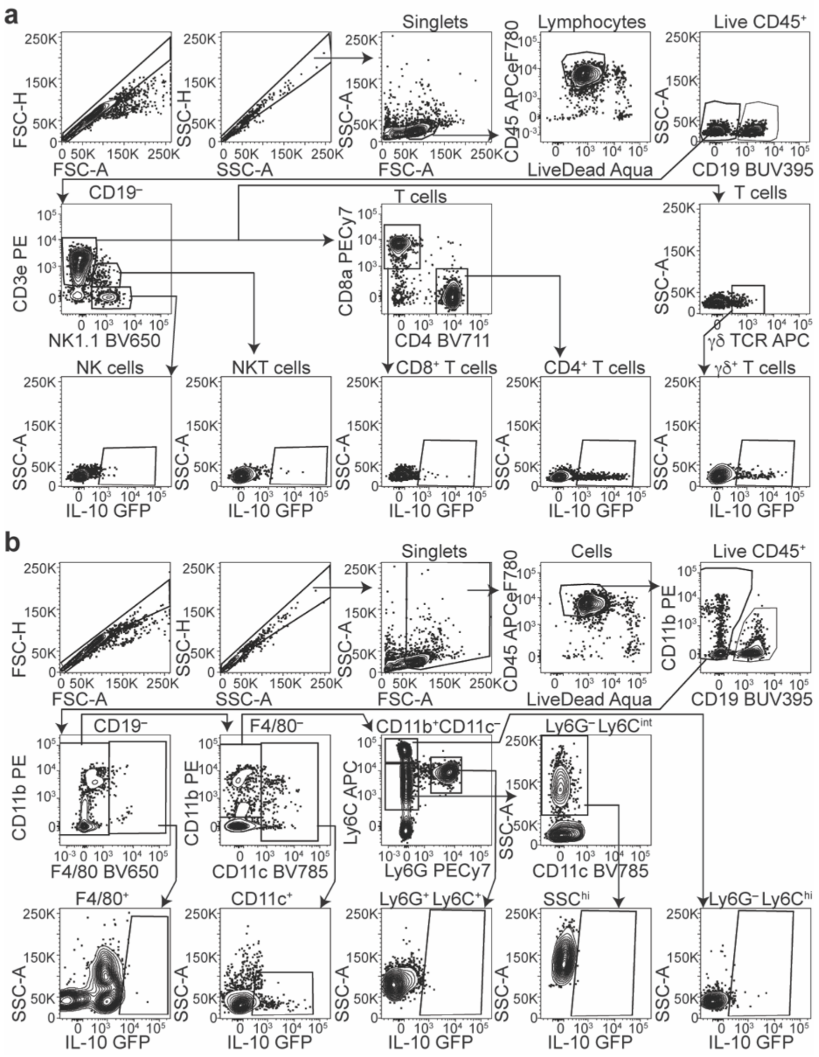

Supplementary Figure 3. Gating strategy for non-B cell leukocytes.

Gating strategy for non-B cell leukocytes; related to Supplementary Figure 2b. (a) Frequency of IL-10^{+} cells based on GFP expression in non-stimulated samples among lymphocyte subsets: pregated on singlets, lymphocyte scatter, LiveDead$^{–}$ CD45^{+} CD19$^{–}$ then subgated for CD4^{+} T cells (CD3^{+}NK1.1$^{–}$CD4^{+}CD8$^{–}$), CD8^{+} T cells (CD3^{+}NK1.1$^{–}$CD4$^{–}$CD8^{+}), γδ T cells (CD3^{+}NK1.1$^{–}$γδ TCR^{+}), NK cells (CD3$^{–}$NK1.1^{+}), NKT cells (CD3^{+}NK1.1^{+}). (b) Frequency of IL-10^{+} cells based on GFP expression in non-stimulated samples among myeloid/granulocyte subsets: pregated on singlets, cell scatter, LiveDead$^{–}$ CD45^{+}CD19$^{–}$ then subgated on macrophages (F4/80^{+}), neutrophils (F4/80$^{–}$CD11b^{+}Ly6G^{+}Ly6C^{+}), inflammatory monocytes

(iMOs; $F4/80^{-}CD11b^{+}Ly6G^{-}Ly6C^{hi}$), eosinophils ($F4/80^{-}CD11b^{+}Ly6G^{-}Ly6C^{lo}$), and dendritic cells ($F4/80^{-}CD11c^{+}$).

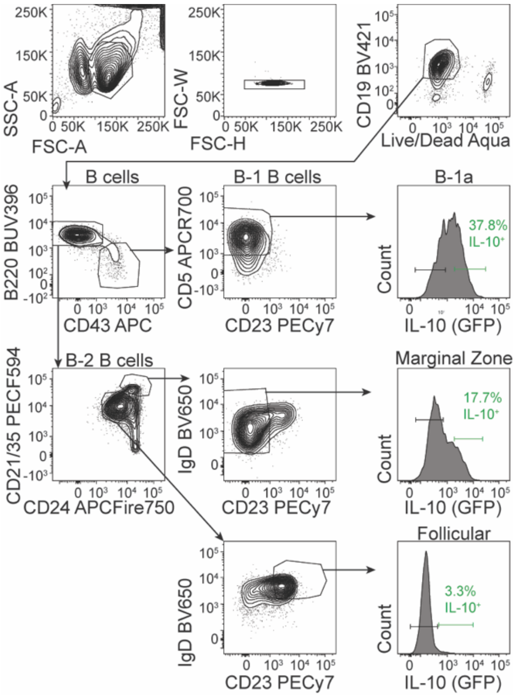

Supplementary Figure 4. FACS sorting gating strategy for IL-10^{+} and IL-10$^{–}$ B-1a, MZ, and FO B cell subsets for clonotype analysis.

Flow cytometric gating strategy for IL-10^{+} (GFP^{+}) and IL-10$^{–}$ (GFP$^{–}$) sIgM$^{–/–}$ B cells based on GFP expression in B-1a, MZ, and FO B cell subsets; related to Figure 3. GFP^{+} cells determined using GFP$^{–}$ sIgM$^{–/–}$ mice. Subsets were gated on singlets, lymphocytes, Live/Dead (L/D) stain$^{–}$, CD45^{+}, CD4^{neg}, CD19^{+}, followed by markers specific to each B cell subset: B-1a: B220$^{lo/–}$ CD43^{+}CD5^{+}; marginal zone (MZ): B220^{hi}CD43$^{–}$CD24^{hi}CD21^{hi}CD23$^{–}$; and follicular (FO): B220^{hi}CD43$^{–}$CD24^{int}CD21^{int}CD23^{+}.

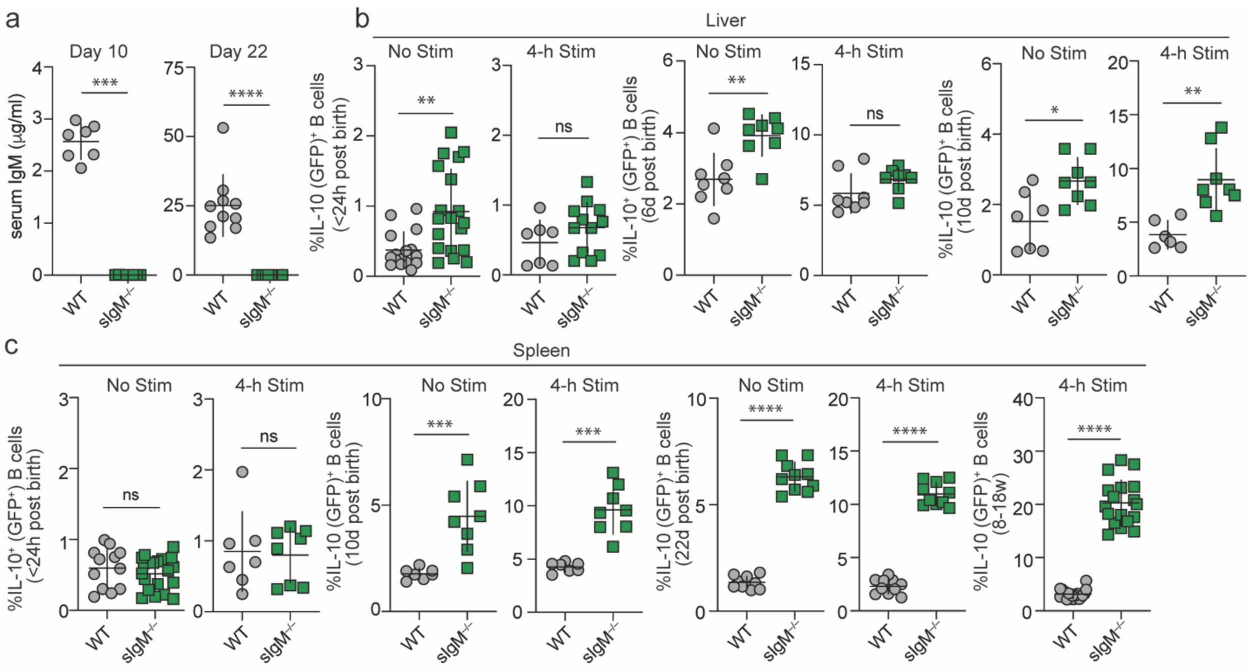

Supplementary Figure 5. The expansion of IL-10$^+$ B cells in the absence of sIgM appears perinatally.

(a) Serum IgM concentration in WT and sIgM$^{-/-}$ mice at day 10 and 22 (weaning age) post birth determined by ELISA (day 10, WT n=7, sIgM$^{-/-}$ n=8, ***p=0.0003; day 22 WT n=10, sIgM$^{-/-}$ n=10, ****p<0.0001). (b) Flow cytometric analysis of liver IL-10$^+$ B cells with and without 4-h stimulation with phorbol 12-myristate 13-acetate/ionomycin/lipopolysaccharide (P/I/L) at <24h, d6, and d10 post birth in WT and sIgM$^{-/-}$ mice (<24h, No Stim: WT n=16, sIgM$^{-/-}$ n=19 **p=0.0018, 4h Stim: WT n=7, sIgM$^{-/-}$ n=12 p=0.0953, ***p=0.0003; day 6, No Stim: WT n=8, sIgM$^{-/-}$ n=8, **p<0.0075, 4h Stim: WT n=8, sIgM$^{-/-}$ n=8 p=0.1848; day 10, No Stim: WT n=7, sIgM$^{-/-}$ n=8 *p=0.0200, 4h Stim: WT n=7, sIgM$^{-/-}$ n=8 **p=0.0013). (c) Flow cytometric analysis of splenic IL-10$^+$ B cells with and without 4-h stimulation with P/I/L at d10 and d22 (weaning) post birth and in adult WT and sIgM$^{-/-}$ mice (<24h, No Stim: WT n=12, sIgM$^{-/-}$ n=19 p=0.3011, 4h Stim: WT n=7, sIgM$^{-/-}$ n=12 p=0.0953, p=0.7789; day 6, No Stim: WT n=7, sIgM$^{-/-}$ n=8, ***p<0.0003, 4h Stim: WT n=8, sIgM$^{-/-}$ n=8 ***p=0.0002; day 10, No Stim: WT n=7,

sIgM$^{-/-}$ n=8 ***p=0.0006, 4h Stim: WT n=7, sIgM$^{-/-}$ n=8 ***p=0.0003; day 22, No Stim: WT n=10, sIgM$^{-/-}$ n=10 ****p<0.0001, 4h Stim: WT n=10, sIgM$^{-/-}$ n=10 ****p<0.0001; 8-18 weeks, No Stim: WT n=27, sIgM$^{-/-}$ n=27 ****p<0.0001, 4h Stim: WT n=18, sIgM$^{-/-}$ n=18 ****p<0.0001). Data points indicate mean ± SD and each symbol represents one mouse. P values were calculated using two-tailed Mann-Whitney U test. Data are pooled from 2 (a; b: 6 and 10 days; c: <24h stim, 10 and 22 days no stim and stim), 3 (b: <24h stim), or 4 (b: <24h no stim; c: <24h no stim) independent experiments. Not significant (ns). Source data are provided with this paper.

Supplementary Table 1. Antibodies used in this study.

**Flow Cytometry**					
**Antibody**	**Fluorophore**	**Company**	**Catalog**	**Clone**	**Dilution**
anti-mouse CD16/32	n/a	BioXCell	2.4G2	2.4G2	1:12
Fixable Live/Dead Fluorescent Dye	Aqua	ThermoFisher	L34966	n/a	1:120
anti-mouse BP-1 (CD249)	PE	ThermoFisher	12-5891-82	6C3	1:25
anti-mouse CD1d	PECy7	Biolegend	123523	1B1	1:400
anti-mouse CD3e	Biotin	eBioscience	13-0031-82	145-2C11	1:100
anti-mouse CD3e	PE	eBioscience	12-0031-82	145-2C11	1:200
anti-mouse CD3e	PECy5	Biolegend	100309	145-2C11	1:200
anti-mouse CD4	BV711	BD Biosciences	563726	RM4-5	1:800
anti-mouse CD5	APC	eBioscience	17-0051-80	53-7.3	1:400
anti-mouse CD5	APCR700	BD Biosciences	565505	53-7.3	1:100
anti-mouse CD5	Biotin	BD Biosciences	553018	53-7.3	1:400
anti-mouse CD5	PE	eBioscience	12-0051-82	53-7.3	1:600
anti-mouse CD8a	PECy7	eBioscience	25-0081-82	53-7.3	1:400
anti-mouse CD9	APC	Biolegend	124810	MZ3	1:100
anti-mouse CD9	FITC	Biolegend	124808	MZ3	1:100
anti-mouse CD11b	PE	eBioscience	12-0112-82	M1/70	1:400
anti-mouse CD11b	Pacific Blue	Biolegend	101224	M1/70	1:200
anti-mouse CD11c	BV785	Biolegend	117335	N418	1:100
anti-mouse CD19	BV421	BD Biosciences	562701	1D3	1:800
anti-mouse CD19	BUV395	BD Biosciences	563557	1D3	1:800
anti-mouse CD19	BV711	BD Biosciences	563157	1D3	1:300
anti-mouse CD19	PE	eBioscience	12-0193-82	1D3	1:400
anti-mouse CD19	Biotin	eBioscience	13-0193-82	1D3	1:100
anti-mouse CD19	AF647	eBioscience	51-0193-82	1D3	1:300
anti-mouse CD21/35	PECF594	BD Biosciences	563959	7G6	1:800
anti-mouse CD23	PECy7	eBioscience	25-0232-82	B3B4	1:200
anti-mouse CD24	APCFire750	Biolegend	101839	M1/69	1:200
anti-mouse CD43	APC	BD Biosciences	560663	S7	1:300
anti-mouse CD43	FITC	BD Biosciences	553270	S7	1:200

anti-mouse CD43	PerCPCy5.5	BD Biosciences	562865	S7	1:200
anti-mouse CD45R/B220	BUV395	BD Biosciences	563793	RA3-6B2	1:200
anti-mouse CD45R/B220	BV421	Biolegend	103240	RA3-6B2	1:200
anti-mouse CD45R/B220	PECy5.5	eBioscience	35-0452-82	RA3-6B2	1:200
anti-mouse CD45R/B220	APCH7	BD Biosciences	565371	RA3-6B2	1:200
anti-mouse CD45	AF350	R&D Systems	FAB114U	30-F11	1:200
anti-mouse CD45	AF700	Biolegend	103128	30-F11	1:200
anti-mouse CD45	APCeF780	eBioscience	47-0451-82	30-F11	1:500
anti-mouse CD45.1	PE	eBioscience	12-0453-82	A20	1:400
anti-mouse CD45.1	eF450	eBioscience	48-0453-82	A20	1:200
anti-mouse CD45.2	PECy5.5	eBioscience	45-0454-82	104	1:200
anti-mouse CD93	BV650	BD Biosciences	563807	AA4.1	1:100
anti-mouse CD138	BV421	BD Biosciences	566289	281-2	1:400
anti-mouse CD138	BV711	BD Biosciences	563193	281-2	1:100
anti-mouse F4/80	Biotin	eBioscience	13-4801-82	BM8	1:100
anti-mouse F4/80	PECy5	Biolegend	123112	BM8	1:100
anti-mouse F4/80	BV650	Biolegend	123419	BM8	1:200
anti-mouse gamma delta TCR	APC	eBioscience	17-5711-82	GL3	1:400
anti-mouse Gr-1	PerCPCy5.5	BD Biosciences	552093	RB6-8C5	1:200
anti-mouse IgD	FITC	eBioscience	11-5993-82	11-26c	1:200
anti-mouse IgD	PE	eBioscience	12-5993-83	11-26c	1:400
anti-mouse IgD	eF450	eBioscience	48-5993-82	11-26c	1:200
anti-mouse IgD	BV650	Biolegend	405721	11-26c	1:800
anti-mouse IgD	AF647	eBioscience	51-5993-82	11-26c	1:400
anti-mouse IgG1	PerCPCy5.5	Biolegend	406612	RMG1-1	1:400
anti-mouse IgG1	PE	Biolegend	406608	RMG1-1	1:800
anti-mouse IgM	PE	eBioscience	12-5790-82	II/41	1:300
anti-mouse IgM	BV650	BD Biosciences	743326	II/41	1:100
anti-mouse IgM	PECy7	eBioscience	25-5790-82	II/41	1:200
anti-mouse IgMa	BUV395	BD Biosciences	743891	DS-1	1:200
anti-mouse IgMa	FITC	BD Biosciences	553516	DS-1	1:200

anti-mouse IgMb	PE	BD Biosciences	553521	AF6-78	1:200
anti-mouse IgMb	BV785	BD Biosciences	742348	AF6-78	1:200
anti-mouse IgMb	Biotin	Biolegend	406204	AF6-78	1:800
anti-mouse IL-10	PE	eBioscience	12-7101-82	JES5-16E	1:100
anti-mouse IL-10	BV421	Biolegend	505022	JES5-16E	1:100
anti-mouse IL-10	APC	eBioscience	554468	JES5-16E	1:100
anti-mouse Ly6C	APC	Biolegend	128016	HK1.4	1:400
anti-mouse Ly6G	PECy7	Biolegend	127618	HK1.4	1:400
anti-mouse NK1.1	Biotin	Biolegend	108703	PK136	1:200
anti-mouse NK1.1	BV650	BD Biosciences	564143	PK136	1:200
anti-mouse TIM-1	BV421	BD Biosciences	566336	RMT1-4	1:100
anti-mouse TIM-1	PE	Biolegend	119506	RMT1-4	1:100
anti-mouse IgG2b	PE	Biolegend	400636	RTK4530	1:100
anti-mouse IgG2b	BV421	Biolegend	400640	RTK4530	1:100
anti-mouse IgG2b	APC	eBioscience	17-4031-82	eB149/10H5	1:100
Streptavidin	PerCPCy5.5	BD Biosciences	551419	n/a	1:200
Streptavidin	BV650	Biolegend	405231	n/a	1:400
Streptavidin	BV785	Biolegend	405249	n/a	1:400
**ELISA**					
**Antibody**	**Fluorophore**	**Company**	**Catalog**	**Clone**	**Dilution**
goat-anti-mouse IgM	n/a	Bethyl Labs	A90-101A	polyclonal	1:100
IgM Standard	n/a	ThermoFisher	39-50470-65	--	as directed by manufacturer (lot specific)
goat-anti-mouse IgM	HRP	Bethyl Labs	A90-101P	polyclonal	1:12,500
IL-10 Mouse Duoset	n/a	R&D Systems	DY417-05	--	as directed by manufacturer (lot specific)
