# Supplementary material for: Secreted IgM modulates IL-10 expression in B cells
Source: Nat Commun. 2024 Jan 5;15:324. doi: 10.1038/s41467-023-44382-w (PMC10773282; doi:10.1038/s41467-023-44382-w)
Supplement: Supplementary file 3 — Description of Additional Supplementary Files [file 41467_2023_44382_MOESM3_ESM.pdf]

Description of Additional Supplementary Files

File Name: Supplementary Data 1
Description: All VJ light chain clonotypes identified from IL-10^{+} and IL-10^{-} B-1a, marginal zone (MZ), and follicular (FO) B cell subsets from spleens of sIgM$^{-/-}$IL-10^{GFP} mice.
