# Supplementary material for: Secreted IgM modulates IL-10 expression in B cells
Source: Nat Commun. 2024 Jan 5;15:324. doi: 10.1038/s41467-023-44382-w (PMC10773282; doi:10.1038/s41467-023-44382-w)
Supplement: Supplementary file 5 — Reporting Summary [file 41467_2023_44382_MOESM5_ESM.pdf]

nature portfolio

Corresponding author(s): Gudrun Debes

Last updated by author(s): Nov 21, 2023

Reporting Summary

Nature Portfolio wishes to improve the reproducibility of the work that we publish. This form provides structure for consistency and transparency in reporting. For further information on Nature Portfolio policies, see our Editorial Policies and the Editorial Policy Checklist.

Statistics

For all statistical analyses, confirm that the following items are present in the figure legend, table legend, main text, or Methods section.

n/a	Confirmed	
☐	☒	The exact sample size (*n*) for each experimental group/condition, given as a discrete number and unit of measurement
☐	☒	A statement on whether measurements were taken from distinct samples or whether the same sample was measured repeatedly
☐	☒	The statistical test(s) used AND whether they are one- or two-sided<br>*Only common tests should be described solely by name; describe more complex techniques in the Methods section.*
☒	☐	A description of all covariates tested
☒	☐	A description of any assumptions or corrections, such as tests of normality and adjustment for multiple comparisons
☐	☒	A full description of the statistical parameters including central tendency (e.g. means) or other basic estimates (e.g. regression coefficient) AND variation (e.g. standard deviation) or associated estimates of uncertainty (e.g. confidence intervals)
☐	☒	For null hypothesis testing, the test statistic (e.g. *F*, *t*, *r*) with confidence intervals, effect sizes, degrees of freedom and *P* value noted<br>*Give P values as exact values whenever suitable.*
☒	☐	For Bayesian analysis, information on the choice of priors and Markov chain Monte Carlo settings
☒	☐	For hierarchical and complex designs, identification of the appropriate level for tests and full reporting of outcomes
☒	☐	Estimates of effect sizes (e.g. Cohen's *d*, Pearson's *r*), indicating how they were calculated

Our web collection on statistics for biologists contains articles on many of the points above.

Software and code

Policy information about availability of computer code

Data collection	Flow cytometry data was acquired on BD LSRFortessa, LSRII, FACSymphony A5, FACSCelesta, or FACS AriaII using BD FACSDiva software (version 8.0.1).<br>qPCR data was acquired using Applied Biosystems StepOnePlus Real-Time PCR System. (StepOne Software version 2.2.2).<br>ELISA data was collected on Berthold, Tristar2 LB942 plate reader using their ICE program (version 1.0.9.8).<br>V(D)J libraries were sequenced on an Illumina NextSeq 500.
Data analysis	Flow cytometry analysis: Data was analyzed using FlowJo (Treestar, version 10), Data was graphed and statistical analysis performed using Graphpad Prism (version 9)<br>ELISA data was analyzed using 4-parameter logistic regression on Graphpad prism (version 9).<br>qPCR data was analyzed using Microsoft Excel (version 16), data was analyzed using housekeeping control gene (Rplp0) and each individual sample was run in duplicate.<br>V(D)J analysis. Raw sequencing reads were processed using the Cell Ranger v5.0 pipeline from 10X Genomics. Sequenced BCL files were converted to fastq using Cellranger 'mkfastq' function, and reads were demultiplexed between the appropriate samples. For V(D)J BCR, cellranger 'vdj' function was used to generate annotated single cell V(D)J sequences for identification of CDR3 sequence and the rearranged V(D)J gene. For annotations, we used GRCm38 version of the mouse genome. Clonotype file from CellRanger was further processed to perform downstream analysis using scRepertoire (doi: 10.12688/f1000research.22139.2) to analyze diversity scores (Shannon and Inverse Simpson). The filtering and plotting was performed using R programming language.

For manuscripts utilizing custom algorithms or software that are central to the research but not yet described in published literature, software must be made available to editors and reviewers. We strongly encourage code deposition in a community repository (e.g. GitHub). See the Nature Portfolio guidelines for submitting code & software for further information.

Data

Policy information about availability of data

All manuscripts must include a data availability statement. This statement should provide the following information, where applicable:

- Accession codes, unique identifiers, or web links for publicly available datasets
- A description of any restrictions on data availability
- For clinical datasets or third party data, please ensure that the statement adheres to our policy

Source data generated in this study are provided in the Supplementary Information/Source Data file. The V(D)J RNA sequencing data generated in this study have been deposited in NCBI GEO under accession number GSE229063 and annotated using reference genome GRCm38, and all VDJ processed data are provided in Supplemental Table 1. All other data are available in the article and its Supplementary Information files or from the corresponding author upon request. Source data are provided with this paper.

Research involving human participants, their data, or biological material

Policy information about studies with human participants or human data. See also policy information about sex, gender (identity/presentation), and sexual orientation and race, ethnicity and racism.

Reporting on sex and gender	N/A
Reporting on race, ethnicity, or other socially relevant groupings	N/A
Population characteristics	N/A
Recruitment	N/A
Ethics oversight	N/A

Note that full information on the approval of the study protocol must also be provided in the manuscript.

Field-specific reporting

Please select the one below that is the best fit for your research. If you are not sure, read the appropriate sections before making your selection.

☒ Life sciences ☐ Behavioural & social sciences ☐ Ecological, evolutionary & environmental sciences

For a reference copy of the document with all sections, see nature.com/documents/nr-reporting-summary-flat.pdf

Life sciences study design

All studies must disclose on these points even when the disclosure is negative.

Sample size	We performed experiments based on previously published studies in our laboratory (Geherin S et al. J Immunol. 2016 Mar 15;196(6):2514-2525; Wilson RP et al, J Invest Dermatol. 2019 Dec;139(12):2477-2487; Aira LE, Debes GF, J Invest Dermatol. 2021 Aug;141(8):1995-2005.e6), and included a total N of 6-27 per group (indicated in each figure legend), except for the VDJ single cell analysis which used 30,000 total input B cells from six populations and targeted 5,000 cells per sub-population (IL-10+ and IL-10– cells from B1a, MZ, and FO B cells subsets) from 1 sIgM-/-IL10GFP mouse and is deemed sufficient in the field to perform downstream analysis (diversity scores). ELISA and qPCR samples were run in duplicate for each sample.
Data exclusions	In repertoire analysis, clonotypes were filtered to remove any sequenced cell that expresses T-cell markers CD3G and CD3D, and we excluded cells expressing IL-10 transcript from IL-10– samples. No other data were excluded from the analyses.
Replication	Experiments were successfully replicated at least two times, specific number of repeats are indicated in each figure legend. VDJ single cell analysis was not repeated in an independent experiment because we analyzed clonality differences based on IL-10 presence within B cell subsets of a mouse. To sufficiently detect statistical differences in diversity between B cell subsets, we targeted 5,000 cells per population from 30,000 total input B cells and analyzed 2,094 - 3,467 cells per sub-population (IL-10+ and IL-10– cells from B1a, MZ, and FO B cells subsets).
Randomization	All mice were grouped by their genotype, and within each group, mice were randomly chosen to be matched in age and sex. For adoptive transfer experiments, recipient mice were matched for age and sex but randomized to which donor genotype they received.
Blinding	For adoptive transfer experiments, recipient mice were randomized to donor genotype, and the investigators were not blinded to allocation during experiments and outcome assessment. For flow cytometry analysis, the same gating strategies were applied equally to all FCS files for each analysis regardless of experimental group.

Reporting for specific materials, systems and methods

We require information from authors about some types of materials, experimental systems and methods used in many studies. Here, indicate whether each material, system or method listed is relevant to your study. If you are not sure if a list item applies to your research, read the appropriate section before selecting a response.

Materials & experimental systems

n/a	Involved in the study
☐	☒ Antibodies
☒	☐ Eukaryotic cell lines
☒	☐ Palaeontology and archaeology
☐	☒ Animals and other organisms
☒	☐ Clinical data
☒	☐ Dual use research of concern
☒	☐ Plants

Methods

n/a	Involved in the study
☒	☐ ChIP-seq
☐	☒ Flow cytometry
☒	☐ MRI-based neuroimaging

Antibodies

Antibodies used

Flow Cytometry
Antibody, Fluorophore, Company, Catalog, Clone, Dilution
anti-CD16/32, n/a, BioXCell ,2.4G2, 2.4G2
Fixable Live/Dead Fluorescent Dye, Aqua, ThermoFisher, L34966 1:120
BP-1 (CD249), PE, ThermoFisher, 12-5891-82, 6C3, 1:25
CD1d, PECy7, Biolegend, 123523, 1B1, 1:400-1:800
CD3e, Biotin, eBioscience, 13-0031-82, 145-2C11, 1:100
CD3, PE, eBioscience, 12-0031-82, 145-2C11, 1:200
CD3e, PECy5, Biolegend, 100309, 145-2C11, 1:200
CD4, BV711 BD Biosciences, 563726, RM4-5, 1:800
CD5, APC, eBioscience, 17-0051-80, 53-7.3, 1:400
CD5, APCR700, BD Biosciences, 565505, 53-7.3, 1:100
CD5, biotin, BD Bioscience, 553018, 53-7.3, 1:400
CD5, PE, eBioscience, 12-0051-82, 53-7.3, 1:600
CD8a, PE-Cy7, eBioscience, 25-0081-82, 53-6.7, 1:400
CD9, APC, Biolegend, 124810, MZ3, 1:100
CD9, FITC, Biolegend, 124808, MZ3, 1:100
CD11b, PE, eBioscience, 12-0112-82, M1/70, 1:400
CD11b, PacBlue ,Biolegend, 101224, M1/70, 1:200
CD11c, BV785, Biolegend, 117335, N418, 1:100
CD19, BV421, BD Biosciences, 562701, 1D3, 1:800
CD19, BUV395, BD Biosciences, 563557, 1D3, 1:800
CD19, BV711, BD Biosciences, 563157, 1D3, 1:300
CD19, PE, eBioscience, 12-0193-82, 1D3, 1:400
CD19, biotin, eBioscience, 13-0193-82, 1D3, 1:100
CD19, AF647, eBioscience, 51-0193-82, 1D3, 1:300
CD21/35, PE-CF594, BD Biosciences, 563959, 7G6, 1:800
CD23, PECy7, eBioscience, 25-0232-82, B3B4, 1:200
CD24, APCFire750, BioLegend, 101839, M1/69, 1:200
CD43, APC, BD Biosciences, 560663, S7, 1:300
CD43, FITC, BD Biosciences, 553270, S7, 1:200
CD43, PerCPCy5.5, BD Biosciences, 562865, S7, 1:200
CD45R/B220, BUV395, BD Biosciences, 563793, RA3-6B2, 1:200
CD45R/B220, BV421, Biolegend, 103240, RA3-6B2, 1:200
CD45R/B220, PECy5.5, eBiosciences, 35-0452-82, RA3-6B2, 1:200
CD45R/B220, APCH7, BD Biosciences, 56537`, RA3-6B2, 1:200
CD45, AF350, R&D Systems, FAB114U, 30-F11, 1:200
CD45, AF700, Biolegend, 103128, 30-F11, 1:200
CD45, APCeF780, eBioscience, 47-0451-82, 30-F11, 1:500
CD45.1, PE, eBioscience, 12-0453-82, A20, 1:400
CD45.1, eF450, eBioscience, 48-0453-82, A20, 1:200
CD45.2 PE-Cy5.5, eBioscience, 45-0454-82, 104, 1:200
CD93, BV650, BDBiosciences, 563807, AA4.1, 1:100
CD138, BV421, BD Biosciences, 566289, 281-2, 1:400
CD138, BV711, BD Biosciences, 563193, 281-2, 1:100
F4/80, biotin, eBioscience, 13-4801-82, BM8, 1:100
F4/80, PECy5, Biolegend, 123112, BM8, 1:100
F4/80, BV650, Biolegend, 123419, BM8, 1:200
g/d TCR, APC, eBioscience, 17-5711-82, GL3, 1:400
Gr-1, PerCPCy5.5, BD Biosciences, 552093, RB6-8C5, 1:200

IgD, FITC, eBioscience, 11-5993-82, 11-26c, 1:200
IgD, PE, eBioscience, 12-5993-83, 11-26c, 1:400
IgD, eF450, eBioscience, 48-5993-82, 11-26c, 1:200
IgD, BV650, Biolegend, 405721, 11-26c, 1:800
IgD, AF647, eBioscience, 51-5993-82, 11-26c, 1:400
IgG1, PerCPCy5.5, Biolegend, 406612, RMG1-1, 1:400
IgG1, PE, Biolegend, 406608, RMG1-1, 1:800
IgM, PE, eBioscience, 12-5790-82, II/41, 1:300
IgM, BV650, BD Biosciences, 743326, II/41, 1:100
IgM, PECy7, eBioscience, 25-5790-82, II/41, 1:200
IgMa, BUV395, BD Biosciences, 743891, DS-1, 1:200
IgMa, FITC, BD Biosciences, 553516, DS-1, 1:200
IgMb, PE, BD Biosciences, 553521, AF6-78, 1:200
IgMb, BV785, BD Biosciences, 742348, AF6-78, 1:200
IgMb, biotin, Biolegend, 406204, AF6-78, 1:800
IL-10, PE, eBioscience, 12-7101-82, JES5-16E, 1:100
IL-10, BV421, Biolegend, 505022, JES5-16E3, 1:100
IL-10, APC, Biolegend, 554468, JES5-16E3, 1:100
Ly6C, APC, Biolegend, 128016, HK1.4, 1:400
Ly6G, PE-Cy-7, Biolegend, 127618, 1A8, 1:400
NK1.1, biotin, Biolegend, 108703, PK136, 1:200
NK1.1, BV650, BD Biosciences, 564143, PK136, 1:200
TIM-1, BV421, BD Biosciences, 566336, RMT1-4, 1:100
TIM-1, PE, Biolegend, 119506, RMT1-4, 1:100
IgG2b, PE, Biolegend, 400636, RTK4530, 1:100
IgG2b, BV421, Biolegend, 400640, RTK4530, 1:100
IgG2b, PE, eBioscience, 17-4031-82, eB149/10H5, 1:100
Streptavidin, PerCPCy5.5, BD Biosciences, 551419 , 1:200
Streptavidin, BV650, Biolegend, 405231, 1:400
Streptavidin, BV785, Biolegend, 405249, 1:400

ELISA
Antibody Fluorophore Company Catalog Clone Dilution
goat-anti-mouse IgM, Bethyl Labs, A90-101A, 1:100
IgM standard, ThermoFisher, 39-50470-65, as directed by manufacturer (lot specific)
goat-anti-mouse IgM, HRP, Bethyl Labs, A90-101P, 1:12,500
IL-10 Mouse Duoset, R&D Systems, DY417-05, as directed by manufacturer

Validation

Antibodies are used from commercial suppliers and are provided with data sheets and validation. Every new flow cytometry antibody is titrated in the lab and validated through its reported staining pattern. New IgM ELISA detection antibodies are validated through titration against a previously assessed lot.

Animals and other research organisms

Policy information about studies involving animals; ARRIVE guidelines recommended for reporting animal research, and Sex and Gender in Research

Laboratory animals

We used mice (Mus musculus) on C57BL/6 background for our studies; Strains used: C57BL/6 (The Jackson Laboratory, stock number 000664), B6.SJL (002014), IgHa congenic (001317), and IL-10GFP (014530). sIgM-/- (received from F. Lund originally and fully backcrossed to C57BL/6 for >10 generations), Blimp-1GFP mice, CD22-/- mice, CMVcreFcmr-/-, CD19cre+/-Fcmrfl/fl. Mice were between 8-18 weeks of age for all experiments except for newborn studies and ages are specified in manuscript. Mice were bred in house. Mice are give ad libitum access to chow and water, have a 12-h/12-h light/dark cycle and are maintained at 20-22 degrees C, 30-70% humidity in specific-pathogen free conditions. Experimental and control mice were bred and caged separately, except for littermate and adoptive transfer studies, in which mice were co-housed.

Wild animals

No wild animals were used in this study.

Reporting on sex

Both male and female mice were used in experiments and matched between groups. Sex was not considered during study design.

Field-collected samples

This study did not include field-collected samples.

Ethics oversight

Animal procedures were approved by the Institutional Care and Use Committees of Thomas Jefferson University (TJU), the Veterans Affairs Palo Alto Health Care System, and the University of California at Davis.

Note that full information on the approval of the study protocol must also be provided in the manuscript.

Plants

Seed stocks

N/A

Novel plant genotypes

N/A

Authentication

N/A

Flow Cytometry

Plots

Confirm that:

☒ The axis labels state the marker and fluorochrome used (e.g. CD4-FITC).

☒ The axis scales are clearly visible. Include numbers along axes only for bottom left plot of group (a 'group' is an analysis of identical markers).

☒ All plots are contour plots with outliers or pseudocolor plots.

☒ A numerical value for number of cells or percentage (with statistics) is provided.

Methodology

Sample preparation

Cells were isolated from mouse spleens, LNs, and newborn livers by grinding tissues with a syringe plunger through 70-um and 40-um cell strainers. Bone marrow was flushed from the femurs using a 25G needle. Red blood cells were lysed from spleen, newborn liver, and bone marrow samples using red cell lysis buffer. Peritoneal cavity cells were obtained by peritoneal lavage with ~9 ml PBS. Single cells from all tissues were washed, counted by hemocytometer and resuspended in complete RPMI (RPMI 1640 with 25mM HEPES, 1X Glutamax, 100 U/mL penicillin, 100 ug/mL streptomycin, 10% newborn calf serum, 50 uM BME).
To analyze cytokine production, cells (1-4x106 cells/ml) were stimulated for 4 hours with 10 ng/ml phorbol 12-myristate 13-acetate (PMA) (Sigma-Aldrich P8139), 0.5 ug/ml ionomycin (Sigma-Aldrich I0634), and 10 ug/ml LPS (Sigma-Aldrich L4641) at 37C 5% CO2 13% O2. After 2h, 10 ug/ml brefeldin-A (Sigma-Aldrich B6542) and 2 uM monensin (BD 554724) were added, except for samples from IL-10GFP mice.
For in vitro B cell culture, CD19 microbeads (Miltenyi 130-121-301) were used following manufacturer's instructions to purify B cells (>98%) from single cell splenic preparations. B cells were plated at 2x106 cells/ml and left untouched or stimulated with soluble 5 ug/ml anti-CD40 (BioXcell BE0016-2) or 1 ug/ml LPS (Sigma-Aldrich L2137) for 3 days, and supernatant was frozen at -80C until analysis by ELISA.
For CD22 and FCMR studies only: Spleens and subiliac lymph nodes from CMVcreFcmr–/–, CD19cre/+Fcmr3fl/fl, and CD22–/– mice as well as sex- and age-matched WT controls were harvested intact in complete (c)RPMI (RPMI 1640 with 25mM HEPES, 1X Glutamax, 100 U/mL penicillin, 100 ug/mL streptomycin, 10% serum, 50 uM BME) and shipped overnight on wet ice from the UC Davis and VA Palo Alto, respectively, for early next-day processing in the Debes Laboratory at TJU. Tissues from sIgM–/– and WT mice housed at TJU were harvested intact in cRPMI and stored overnight at 4C for parallel analysis with knockout tissues.
To stain cells for flow cytometry, cells were pre-incubated with mouse anti-CD16/CD32 (2.4G2; Bio X Cell) and rat IgG (Jackson Immunoresearch Laboratories 012-000-003) to reduce non-specific staining and Live/Dead Fixable Aqua Dead Cell Stain (ThermoFisher L34966) to exclude dead cells from analysis. Cells were then stained with the antibodies as detailed above and in Supplemental Table 2. All staining and wash steps were performed at 4C in PBS with 0.2% BSA (Sigma-Aldrich A9418). IL-10GFP reporter samples were immediately acquired on flow cytometers, and samples from non-reporter mice were fixed with 2% paraformaldehyde (PFA; Sigma-Aldrich 158127). Intracellular IL-10 staining was performed on fixed cells using buffer containing 0.5% saponin (Sigma-Aldrich 47036).
To FACS sort for single cell RNAseq, total splenocytes from sIgM–/–IL-10GFP mice were stimulated at 4x106 cells/ml with 10 ng/ml PMA, 0.5 ug/ml Ionomycin, and 10 ug/ml LPS for 4-h at 37C, 5% CO2, 13% O2. B cells were enriched using CD19 microbeads (Miltenyi 130-121-301) following the manufacturer's protocol. Cells were blocked with Fc block and stained for viability and B cell subset markers, washed and sorted on a FACSAria II. Cells were gated on MZ B cells, FO B cells, and B-1 B cells as described above with additional use of CD5 expression to delineate B-1a B cells. Each subset was further sorted into IL-10+ (GFP+) and IL-10– (GFP-) as shown in Supplemental Figure 4. On each sorted subset, cell viability was confirmed using a Countess II FL Automated Cell Counter (ThermoFisher).

Instrument

Flow cytometry data was acquired at Thomas Jefferson University on BD LSRFortessa, LSRII, FACSymphony A5, or Celesta. Cells were sorted on a FACSAriaII.

Software

Flow cytometry data was collected with BD FACSDiva (version 8.0.1). Data was analyzed with BD FlowJo (Version 10), and graphed using Graphpad prism (version 9).

Cell population abundance

Flow cytometry was performed on single cell isolates from tissues (spleen, lymph nodes, bone marrow, liver). The percentage

Cell population abundance	of cells and total number of cells are indicated in the data. For the FACS sorted populations for VDJ analysis, the percentage of IL-10+ cells from each B cell subset B1a, MZ, and FO is indicated in supplemental figure 3. Samples were sorted at >99% purity. The total number of cells sorted on the AriaII for each subset was: B1aPOS: 64,190; B1aNEG: 56,430; MzPOS:67,540; MzNEG:95,500; FoPOS:84,650; FoNEG:154,200. Cells were submitted to TJU Genomics core for viability reassessment using a Countess III FL Automated Cell Counter (ThermoFisher) and capture of 5,000 cells targets for each sample.
Gating strategy	All samples were pre-gated on singlets (FSCAxFSCH then SSCAxSSCH), lymphocyte scatter, Live/Dead Aqua—CD45+ cells. B cells were identified as CD19+ distinguishing B-1 cells as B220lo/negCD43+ with CD5+ (B-1a) and CD5—(B-1b) and B-2 cells as B220hiCD43neg cells. B-2 cells were further distinguished as transitional stage 1 (T1): CD24hiCD21lo/neg; transitional stage 2 (T2): CD24hiCD21int; transitional stage 2-marginal zone precursor (T2-MZP): CD24hiCD21hi, CD23+; marginal zone (MZ): CD24hiCD21hiCD23neg; and follicular (FO): CD24intCD21intCD23+IgD+. Non B cell subsets were identified as lymphocyte subsets: pregated on singlets, lymphocyte scatter, LiveDead– CD45+ CD19– then subgated for CD4+ T cells (CD3+NK1.1–CD4+CD8–), CD8+ T cells (CD3+NK1.1–CD4–CD8+), gd T cells (CD3+NK1.1–gd TCR+), NK cells (CD3–NK1.1+), NKT cells (CD3+NK1.1+), and myeloid/granuolocyte subsets: pregated on singlets, cell scatter, LiveDead– CD45+CD19– then subgated on macrophages (F4/80+), neutrophils (F4/80–CD11b+Ly6G+Ly6C+), inflammatory monocytes (iMOs; F4/80–CD11b+Ly6G–Ly6Chi), eosinophils (F4/80–CD11b+Ly6G–Ly6Clo), and dendritic cells (F4/80–CD11c+).<br>Breg phenotype was identified by expression of TIM-1+ (c), CD1dhiCD5+ (d), or CD9+ (e) among total splenic B cells gated as singlets (FSCA/FSCH), lymphocyte scatter, Live/Dead Aqua—CD19+.<br>Bone Marrow: Cells were gated on singlets (FSCAxFSCH then SSCAxSSCH), Live/Dead (L/D) stain–, CD3–, F4/80–, NK1.1–, Gr-1–, B220+. B220+ BM cells were further subdivided into Hardy fractions: Fr. A: B220+CD43+IgM–BP1–CD24–; Fr. B: B220+CD43+IgM–BP1–CD24+; Fr. C-C': B220+CD43+IgM–BP1+CD24+; Fr. D: B220+CD43–IgM–IgD–; Fr. E: B220+CD43–IgM+IgD–; and Fr. F: B220+CD43–IgM+/–IgD+.<br>Plasma cell identity was assessed by flow cytometry directly ex vivo on singlets (FSCAxFSCH then SSCAxSSCH), L/D stain–, CD45+, CD3-, F4/80–, IgD– and then assessed for Blimp1GFP expression in comparison to Blimp1GFP negative animals.<br>Allelic exclusion of IgMa and IgMb was determined after gating for B cells as above. Control mice expressing only IgMa or IgMb were used to determine gates. IgMa/IgMb fluorophores were switched between experiments to ensure that any differences detected were not due to fluorophore choice.<br>IL10GFP gating was always determined in comparison to GFP negative animals.<br>IL10 protein gating was always determined using appropriate isotype controls.<br>All other markers were identified as positive vs negative based on staining intensity of two populations and/or FMO/isotype controls.

☒ Tick this box to confirm that a figure exemplifying the gating strategy is provided in the Supplementary Information.
